# Greater psychological response and participation in knee‐strenuous activity 8 months after anterior cruciate ligament reconstruction in patients with generalised joint hypermobility who sustained a second anterior cruciate ligament injury: A cross‐sectional registry study

**DOI:** 10.1002/jeo2.70351

**Published:** 2025-07-13

**Authors:** Jakob Lindskog, Axel Sundberg, Johan Högberg, Rebecca Hamrin Senorski, Ramana Piussi, Kristian Samuelsson, Roland Thomeé, Eric Hamrin Senorski

**Affiliations:** ^1^ Unit of Physiotherapy, Department of Health and Rehabilitation, Institute of Neuroscience and Physiology, Sahlgrenska Academy University of Gothenburg Gothenburg Sweden; ^2^ Sahlgrenska Sports Medicine Center, Sahlgrenska Academy Gothenburg Sweden; ^3^ Department of Orthopaedics, Institute of Clinical Sciences, Sahlgrenska Academy University of Gothenburg Gothenburg Sweden; ^4^ Department of Orthopaedics Sahlgrenska University Hospital Mölndal Sweden

**Keywords:** anterior cruciate ligament, ACL reconstruction, generalised joint hypermobility, re‐injury, risk factors, physical evaluation, psychological evaluation

## Abstract

**Purpose:**

To compare patients with generalised joint hypermobility (GJH) who sustained a second anterior cruciate ligament (ACL) injury with those who did not sustain a second ACL injury, in terms of muscle strength, psychological response and level of knee‐strenuous activity after primary ACL reconstruction. We hypothesised that patients who sustained a second ACL injury would display similar muscle strength symmetry, report similar psychological response and report greater levels of knee‐strenuous activity compared to patients who did not sustain a second ACL injury.

**Methods:**

Data from a rehabilitation specific registry were extracted. Patients 15–30 years old with GJH, who sustained a second ACL injury after primary ACL reconstruction were matched 1:2 with patients with GJH who did not sustain a second ACL injury. Outcomes were compared at the 10‐week, 4‐, 8‐ and 12‐month follow‐ups after primary ACL reconstruction. Outcomes comprised limb symmetry index and peak torque relative to body weight for quadriceps and hamstrings muscle groups assessed seated isokinetically with a Biodex. In addition, the questionnaires knee self‐efficacy scale_18_, ACL‐return to sport after injury scale and Tegner activity scale (Tegner) were analysed. All outcome data were analysed with the independent *t*‐test, except for the Tegner which was analysed with the Mann–Whitney *U*‐test.

**Results:**

Thirty‐two patients sustained a second ACL injury and were matched with 64 patients who did not. The mean age at time of ACL reconstruction was 19 ± 3 years, and 58 (60%) were female. There were no differences in muscle strength between groups at any follow‐up. At the 8‐month follow‐up, patients with a second ACL injury reported higher on ACL‐return to sport after Injury (65.6 ± 17.9 vs. 53.5 ± 17.3, *p* = 0.006), and participated in gknee‐strenuous activity (median Tegner 5.0, interquartile range [IQR]: 3.0 versus 4.0, IQR: 4.0, *p* = 0.046) compared to patients without a second ACL injury.

**Conclusion:**

Patients with GJH who sustained a second ACL injury reported greater confidence, lower negative emotions, and lesser risk appraisal towards return to sport, and reported greater levels of knee‐strenuous activity at 8 months after primary ACL reconstruction compared to matched patients who did not sustain a second ACL injury. These findings might suggest that patients who report greater psychological readiness to RTS, that is, greater confidence, lower negative emotions and lesser risk appraisal, at 8 months, need more cautious guidance when increasing knee‐strenuous activity.

**Level of Evidence:**

Level IV, cross‐sectional study.

**Abbreviations**
ACL: anterior cruciate ligament
ACL‐RSI: an
  terior cruciate ligament‐Return to sport after injury
BPTB: bone‐patellar tendon‐bone
CI: confidence interval
cm: centimeters
GJH: generalised joint hypermobility
HE: hyperextension
HT: hamstring tendon
ICC: intraclass correlation coefficient
IQR: interquartile range
kg: kilogram
K‐SES_18_
: 18‐item version of knee self‐efficacy scale
LSI: limb symmetry index
MDC: minimal detectable change
MIC: minimal important change
n: number of patients
PROs: patient reported outocmes
RECORD: REporting of studies Conducted using Observational Routinely‐collected health Data
RTS: return to sport
SD: standard deviation
Tegner: Tegner activity scale

## INTRODUCTION

Rehabilitation after anterior cruciate ligament (ACL) reconstruction aims to recover muscle function, improve symptoms and reduce limitations during physical activity [24]. Among patients who successfully return to sport (RTS), up to 22% in certain subgroups of patients experience a second ACL injury, regardless of sex [25]. The risk for a second ACL injury is especially high for patients with the generalised joint hypermobility (GJH) phenotype [38, 45]. The GJH condition leads to hyperextensibility of synovial joints [28], which can increase the stress on the ACL in cutting or pivoting motions. Since GJH is a nonmodifiable risk factor for a second ACL injury, and to date, there are no guidelines to minimise the risk, further exploration of modifiable risk factors is warranted.

Beyond the nonmodifiable risk factor GJH for a second ACL injury, the risk for a second ACL injury is thought to be modifiable by certain factors including, but not limited to; (1) delay time of RTS to 9–12 months after ACL reconstruction [5, 14], (2), achieve sufficient muscle strength, that is, ≥90% of quadriceps strength compared to the uninjured limb [15] and (3), be psychologically prepared for RTS, that is, score high on the ACL‐return to sport index (ACL‐RSI) scale [23]. However, higher scores on ACL‐RSI and the knee self‐efficacy scale_18_ (K‐SES_18_) have also been reported in patients who sustained a second ACL injury compared to patients who did not [26]. Taken together, the relationship between psychological response and the risk of a second ACL injury appears to be increased by both strong and weak psychological responses. Therefore, evaluation of psychological response, specifically knee‐related self‐efficacy, emotions, confidence and risk appraisal towards RTS, is warranted due to their potential impact on the risk of a second ACL injury. However, to determine the risk of second ACL injury, clinicians must also account for additional factors alongside psychological outcomes. Specifically, they should consider the patient's recovery of muscle function and the intended level of physical activity, as these directly influence knee strain, consequently affecting the overall risk of a second ACL injury [3, 15]. As individuals with GJH constitute a vulnerable population for ACL injury, there is a need to better understand how muscle strength, psychological response and level of knee‐strenuous activity may be related to a second ACL injury.

The aim of this study was therefore to compare muscle strength, psychological response and knee‐strenuous activity during the first 12 postoperative months in patients with GJH who sustained a second ACL injury with patients who did not sustain a second ACL injury within 36 months after primary ACL reconstruction.

We hypothesised that patients who sustained a second ACL injury would display similar muscle strength symmetry, report similar psychological response and report greater levels of knee‐strenuous activity compared to patients who did not sustain a second ACL injury.

## METHODS

### Study design

The present study was conducted as a matched study with cross‐sectional comparisons, structured according to the REporting of studies Conducted using Observational Routinely‐collected health Data (RECORD) statement [7]. Ethical approval has been obtained from the Swedish Ethical Review Authority (registration number: 2020‐02501), and the Regional Ethical Review Board in Gothenburg, Sweden (registration numbers: 265–13, T023–17).

### Setting

Data for the present study were extracted from a rehabilitation specific registry, Project ACL, which has previously been described in detail [16, 17]. Project ACL is located in Gothenburg, Sweden, started in 2014 and aimed to improve the care for patients who have sustained an ACL injury. Patients registered in Project ACL were invited to participate in muscle strength tests guided by an experienced test leader and answer online based questionnaires at predefined follow‐ups at 10 weeks, 4, 8, 12, 18, 24 months, 5 years and then every 5th year after ACL injury/reconstruction. Upon registration in Project ACL, patients were required to provide demographic information such as date of injury, body weight and height. However, data on concomitant injuries or additional surgical procedures are not registered. At the time of muscle strength tests, regardless of follow‐up, the assessment of Beighton Score was performed by the test leader and documented in the Project ACL database.

### GJH

Generalised joint hypermobility was defined based on age and sex, using the Beighton Score [4]. Patients ≥16 years old, with a score of ≥4 for males and ≥5 for females were classified as GJH. Furthermore, patients between 15 and 16 years, with a score of ≥5 for males and ≥6 for females were classified as GJH.

### Participants

Patients registered in Project ACL were checked for eligibility for inclusion by having a primary ACL injury treated with ACL reconstruction using hamstring tendon (HT) or bone‐patellar tendon‐bone (BPTB) autograft, had an age between 15 and 30 years at the time of primary ACL reconstruction, had GJH, participated in knee‐strenuous sports preinjury, that is, scoring ≥6 on Tegner activity scale (Tegner) [35], and had follow‐up data on any of the 10‐week, 4‐, 8‐ and 12‐month follow‐ups.

Patients were excluded from this study if a second ACL injury occurred later than 36 months after primary ACL reconstruction. Since the RTS can take up to 24 months after primary ACL reconstruction [1, 2] and is associated with increased risk for a second ACL injury [3], the time frame of 36 months after primary ACL reconstruction was chosen to account for adequate risk exposure.

Upon inclusion, each patient who sustained a second ACL injury, that is, graft rupture or contralateral ACL injury confirmed clinically or with imaging and registered by the responsible clinician or the patient itself, was matched 1:2 with patients who did not sustain a second ACL injury and had at least 36 months of follow‐up after ACL reconstruction. Matching was performed by utilisation of a propensity score with the K‐nearest neighbour technique. The propensity score was estimated with logistic regression based on the following covariates: patient sex, age at primary ACL reconstruction, but not younger than 15 years or older than 30 years, preinjury Tegner, but not <6, and graft choice for primary ACL reconstruction.

### Second ACL injury

In this study, both graft rupture and contralateral ACL injury were considered as second ACL injury.

### Beighton score

The Beighton score [4] has often been recommended to assess GJH [10], and has inter‐ and intrarater reliability equaling substantial to excellent for raters regardless of expertise [8]. The Beighton Score measures joint mobility on a nine‐point scale, where the eight unilateral tests consist of dorsiflexion of the metacarpal joint of the fifth finger beyond 90°, apposition of the thumbs to the flexor aspects of the forearms, hyperextension of the elbows and knees beyond 10°, and the one bilateral test is forward flexion of the trunk, with the knees straight, so that the palms of the hands are resting easily on the floor. As per recommendation, the use of an injury allowance point was applied to account for possible movement impairment of the involved knee [33]. For patients in pubertal age up to 50 years, a Beighton Score of ≥4 for males, and ≥5 for females is defined as having GJH [22]. For patients in prepubertal age, a Beighton score ≥5 for males, and ≥6 for females is defined as having GJH [22]. In Project ACL the Beighton Score was performed during the first physical evaluation follow‐up by the test leader and entered into the database as a total score. Alongside the total Beighton score, the presence of knee hyperextension (HE), that is, passive hyperextension of the knees beyond 10° was entered as yes/no. Test leaders in Project ACL were thoroughly trained in standardised Beighton score assessment, and are not exclusively affiliated with the present study.

### Variables

#### Tests of muscle strength

The procedure of muscle strength tests in Project ACL was performed according to a predefined protocol previously described in detail [18], and comprise two sections: (1) quadriceps and hamstring muscle strength and (2) hop tests which include vertical hop, hop for distance and 30‐second side hop. In this study, only muscle strength tests were used for analysis. The quadriceps and hamstring muscle strength was assessed with an isokinetic dynamometer, Biodex System 4 (Biodex Medical Systems) [39] in a seated position at an angular velocity of 90°/s. The Biodex dynamometer has near perfect instrumental validity [12], (intraclass correlation coefficient [ICC] = 0.99–1.00) and excellent test‐retest reliability [13] (ICC = 0.95) when measuring peak torque in quadriceps and hamstring strength. The highest achieved torque was registered in the database and used for analysis.

Muscle strength tests were evaluated using (1) the limb symmetry index (LSI) assessed by $\left(\frac{\mathrm{Result}\;\mathrm{for}\;\mathrm{injured}\;\mathrm{leg}}{\mathrm{Result}\;\mathrm{for}\;\mathrm{uninjured}\;\mathrm{leg}}\right)*100$, presented as a percentage, and (2) peak torque relative to body weight assessed by $\left(\frac{\mathrm{Result}\;\mathrm{for}\;\mathrm{injured}\;\mathrm{leg}}{\mathrm{Body}\;\mathrm{weight}\;(\mathrm{kg})}\right)$, presented as a quota.

#### Patient reported outcomes (PROs)

At the time of administration of PROs, patients were first queried on whether they had sustained a second ACL injury. If they had, they could not proceed with completing the PROs.

#### Psychological response

This study used the K‐SES_18_ which aimed to evaluate knee‐related self‐efficacy, that is, the patient's belief in performing a physical knee‐related task, in patients with ACL injury and consisted of 18 items [6]. The scale comprised two subscales, present (14 items) and future (4 items) [6]. Each item was scored between 0 and 10, where 0 represents poor self‐efficacy and 10 excellent self‐efficacy. The score was presented as a mean value for each subscale [6]. The K‐SES_18_ has sufficient construct validity tested as structural validity, hypothesis testing and cross‐cultural adaptation [6]. Test–retest reliability has been reported with an ICC = 0.92, and internal consistency with a Cronbach's *α* = 0.81–0.96 [6]. To further evaluate psychological response, the ACL‐RSI was used [20]. The ACL‐RSI aimed to evaluate emotions, confidence and risk appraisal towards RTS [20]. The present study used the 12‐item version [40]. The items were scored between 1 and 10, normalised to a score between 10 and 100, where 100 represented the greatest confidence, lowest negative emotion, and lowest risk appraisal in relation to RTS [20, 41]. The ACL‐RSI has excellent internal consistency with a Cronbach's *α* = 0.95, relevant face validity tested from expert and patient discussions, good construct validity tested with hypothesis testing [20] and a fair to good ability to predict RTS [20, 42]. The minimal important change (MIC) for the ACL‐RSI has been reported to 2.6 [32].

#### Knee‐strenuous activity

The Tegner is a self‐reported questionnaire which aimed to quantify the patient's level of knee‐strenuous activity. The Tegner comprised work and sport activities. The Tegner used in Project ACL ranged between 1 to 10, where 10 represented the highest knee‐strenuous activity such as professional soccer. The Tegner used in Project ACL was a modified scale where level 0 that represent ‘sick leave or disability pension because of knee problems’, was removed. Tegner level 6 was the first level which only contains sports activity, such as badminton, tennis, or alpine skiing. Tegner ≥6 was considered knee‐strenuous activities and was used to classify patients who performed knee‐strenuous activity activities prior to their primary ACL injury or had RTS at follow‐up. The Tegner has shown test–retest reliability of ICC = 0.8 [36]. The minimal detectable change (MDC) for the Tegner is 1 [9].

#### Data collection

Demographical data were extracted from Project ACL for analysis on 2025‐03‐14, and comprised of categorical data such as sex, graft choice for primary ACL reconstruction, Beighton Score and presence of knee HE. Continuous data for age, height, weight, time between primary ACL injury and surgery, follow‐up time after ACL reconstruction, results of tests of muscle strength and PROs for the 10‐week, 4‐, 8‐ and 12‐month follow‐ups were also extracted. The ACL‐RSI was completed from the 8‐month follow‐up and onwards, while the other questionnaires included were completed from 10 weeks and onwards.

#### Definition of study groups

The first study group comprised patients with GJH who sustained a second ACL injury within 36 months after primary ACL reconstruction and was defined as the ‘second ACL’ group. The second study group comprised matched patients with GJH who did not have a registered second ACL injury at time of data extraction after primary ACL reconstruction and was defined as the ‘control’ group.

#### Outcomes

The primary outcomes of this study were the tests of muscle strength, presented as LSI and peak torque relative to body weight. The secondary outcomes were the assessment of psychological response with the PROs: K‐SES_18_ and ACL‐RSI and and Tegner for assessment of level of knee‐strenuous activity. Tegner was assessed both continuously, and dichotomised RTS (Tegner ≥ 6). All outcomes were extracted for the follow‐ups: 10 weeks, 4, 8, 12 months after primary ACL reconstruction.

#### Statistical analyses

Statistical analyses were performed with the Statistical Product and Service Solutions (IBM Corp. Released 2017. IBM SPSS Statistics for Windows, Version 25.0. Armonk, NY: IBM Corp.). Analyses were carried out and presented stratified by defined groups (second ACL and controls) across the 10‐week, 4‐, 8‐ and 12‐month follow‐ups. Comparison of outcomes was presented as mean ± standard deviation (SD) and analysed with the independent *t*‐test between groups for all outcomes of interest except comparison of Tegner which was presented as median and interquartile range (IQR) and analysed with the Mann–Whitney *U*‐test for the continuous score, and for the dichotomous RTS (Tegner ≥ 6) classification, presented as numbers with percent, and analysed with the Fisher's Exact test. In addition, the Cohen's *d* was calculated for the comparison of parametric data and interpreted as follows; 0.00 < 0.20 as very small, 0.20 < 0.50 as small, 0.50 < 0.80 as medium and ≥0.80 as large [34]. Demographical data were analysed with independent *t*‐test for parametric continuous data, Mann–Whitney *U*‐test for ordinal data and Fisher's Exact test for categorical data for the total included cohort and for each follow‐up. Visual assessments of distribution of data using histograms were used to determine the appropriate choice of statistical test. Continuous data were presented as mean ± SD for parametric data and median with IQR for nonparametric data. Categorical data were presented as numbers with percentage. To account for attrition bias, sensitivity analyses that compared demographics between patients that contributed with data for tests of muscle function and PROs with patients who dropped out from analysis were carried out for all outcomes and all follow‐ups. Based on a power analysis for the primary outcome, that is, LSI of strength tests, assuming a 9.4% difference [15], with a power of 80%, a significance value of 0.05, and an enrolment ratio of 1:2, a total of 51 patients (second ACL group *n* = 17 and controls *n* = 34) was needed. Significance level was set at 0.05. All data were handled and analysed by the first author.

## RESULTS

**Figure 1:**
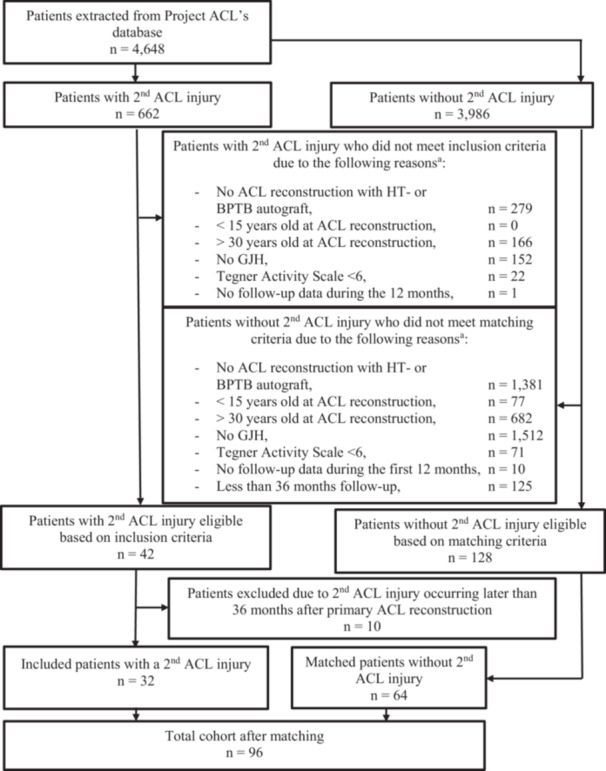
Flowchart of inclusion process. ^a^Inclusion criteria were applied sequentially, and patients were able to fulfill multiple criteria. ACL, anterior cruciate ligament; BPTB, bone‐patellar tendon‐bone; GJH, generalised joint hypermobility; HT, hamstring tendon; *n*, number of patients.

Figure 1 presents the inclusion process where a total of 4648 patients from Project ACL's database were available for eligibility screening for the study, of whom 32 patients had sustained a second ACL injury (27/32, 84% graft rupture, and 5/32, 16% contralateral ACL rupture) and were included. The 32 patients who sustained a second ACL injury were matched with 64 patients who did not sustain a second ACL injury. The mean age of the entire cohort, both patients with second ACL injury and the control group, was 19 ± 3 years and comprised 58 (60%) females. Table 1 presents demographic data for patients who provided data for each of the 10‐week, 4‐, 8‐, 12‐month follow‐ups, separately. There was no difference for demographic variable between groups for each follow‐up.

**Table 1:** Demographical data on included patients in each follow‐up.

Follow‐up	10‐week			4‐month			8‐month				12‐month	
Patient demographics	Second ACL	Controls	*p* value	Second ACL	Controls	*p* value	Second ACL	Controls	*p* value	Second ACL	Controls	
	*n* = 20	*n* = 44		*n* = 25	*n* = 48		*n* = 26	*n* = 56		*n* = 20	*n* = 50	*p* value
Patient sex, females, *n* (%)	12 (60)	31 (71)	0.566	15 (60)	31 (65)	0.800	15 (58)	36 (64)	0.369	11 (55)	32 (64)	0.589
Age, years mean ± SD	20 ± 3	20 ± 3	0.795	19 ± 3	20 ± 3	0.905	19 ± 3	19 ± 3	0.997	19 ± 3	20 ± 3	0.815
Height, cm mean ± SD	173 ± 7	170 ± 8	0.735	174 ± 8	173 ± 8	0.508	174 ± 9	173 ± 10	0.680	174 ± 9	173 ± 10	0.747
Weight, kg mean ± SD	70 ± 9	70 ± 12	0.934	70 ± 9	69 ± 11	0.558	72 ± 10	69 ± 12	0.301	72 ± 10	69 ± 12	0.323
Time from injury to ACL reconstruction, months median (IQR)	3.3 (3.4)	3.4 (3.4)	0.744	3.4 (3.3)	3.5 (3.5)	0.542	3.5 (3.7)	3.5 (3.3)	0.588	4.0 (3.4)	3.4 (3.5)	0.799
Follow‐up time from ACL reconstruction, months median (IQR)	14.3 (10.4)	66.4 (24.5)	<0.001	13.7 (7.8)	68.8 (24.1)	<0.001	13.7 (7.4)	69.4 (33.3)	<0.001	15.3 (7.4)	73.1 (24.9)	<0.001
Knee HE, *n* (%) [*n* missing]	13 (65) [0]	34 (79) [1]	0.351	17 (68) [0]	35 (75) [1]	0.589	18 (72) [1]	41 (76) [2]	0.783	13 (65) [0]	40 (82) [1]	0.207
Preinjury Tegner, median (IQR)	8.5 (1.0)	9.0 (2.0)	0.450	9.0 (2.0)	9.0 (1.0)	0.544	9.0 (1.0)	9.0 (1.0)	0.719	8.5 (1.0)	9.0 (1.0)	0.986
Graft choice, *n* (%)												
HT	18 (90)	40 (91)	1.000	23 (92)	44 (92)	1.000	24 (92)	52 (93)	1.000	18 (90)	47 (94)	0.619
BPTB	2 (10)	4 (9)		2 (8)	4 (8)		2 (8)	4 (7)		2 (10)	3 (6)	
Patients sustaining a second ACL injury between the prior follow‐up to the current follow‐up												
Graft rupture, *n* (%)	0	‐	‐	0	‐	‐	2 (100)	‐	‐	8 (100)	‐	‐
Contralateral ACL injury, *n* (%)	0	‐	‐	0	‐	‐	0	‐	‐	0	‐	‐
Patients sustaining a second ACL injury after the follow‐up up to the next follow‐up												
Graft rupture, *n* (%)	0	‐	‐	2 (100)	‐	‐	8 (100)	‐	‐	17 (77)	‐	‐
Contralateral ACL injury, *n* (%)	0	‐	‐	0	‐	‐	0	‐	‐	5 (23)	‐	‐

Abbreviations: ACL, anterior cruciate ligament; BPTB, bone‐patellar tendon‐bone; cm, centimeters; GJH, generalised joint hypermobility; HE, hyperextension; HT, hamstrings tendon autograft; IQR, interquartile range; kg, kilogram; *n*, number of patients; SD, standard deviation; Tegner, Tegner activity scale.

### Primary outcomes

Table 2 presents the outcomes for muscle strength tests where no differences between groups were found in quadriceps and hamstring strength, for neither LSI nor relative peak torque, at any of the 10‐week, 4‐, 8‐ and 12‐month follow‐ups.

**Table 2:** Cross‐sectional comparisons of tests of muscle strength and patient‐reported outcomes during the first 12 postoperative months between patients with GJH who sustained a second ACL injury and controls who did not after primary ACL reconstruction.

Follow‐up	10 weeks				4 months				8 months				12 months			
Variable	Second ACL	Control	*p* value	Cohen's *d*	Second ACL	Control	*p* value	Cohen's *d*	Second ACL	Control	*p* value	Cohen's *d*	Second ACL	Control	*p* value	Cohen's *d*
	*n* = 32	*n* = 64			*n* = 32	*n* = 64			*n* = 32	*n* = 64			*n* = 32	*n* = 64		
Quadriceps strength, mean ± SD 95% CI [*n*]																
LSI	74.3 ± 14.6	71.0 ± 14.4			87.1 ± 15.2	81.8 ± 13.8			96.0 ± 12.8	93.5 ± 8.8			101.4 ± 16.0	97.0 ± 9.8		
	67.3–81.4	66.2–75.8	0.419	0.23	80.8–93.4	77.4–86.1	0.149	0.37	90.7–101.2	90.9–96.0	0.326	0.24	93.7–109.1	93.9–100.0	0.187	0.37
	[19]	[37]			[25]	[41]			[25]	[49]			[19]	[42]		
Relative	2.1 ± 0.5	2.0 ± 0.5			2.5 ± 0.5	2.4 ± 0.6			2.8 ± 0.5	2.8 ± 0.4			3.0 ± 0.4	2.9 ± 0.5		
	1.9–2.3	1.8–2.1	0.228	0.34	2.3–2.7	2.2–2.5	0.371	0.23	2.6–3.0	2.7–2.9	0.753	0.08	2.8–3.2	2.7–3.0	0.271	0.31
	[19]	[37]			[25]	[41]			[25]	[49]			[19]	[42]		
Hamstrings strength, mean ± SD 95% CI [*n*]																
LSI	82.5 ± 9.8	80.6 ± 17.5			91.7 ± 10.4	92.1 ± 15.0			92.3 ± 9.4	96.0 ± 11.2			96.9 ± 9.2	97.4 ± 12.4		
	77.8–87.2	74.6–86.5	0.658	0.13	87.4–96.0	87.3–96.8	0.921	0.03	88.4–96.1	92.8–99.2	0.153	0.35	92.5–101.3	93.6–101.3	0.860	0.05
	[19]	[36]			[25]	[40]			[25]	[49]			[19]	[42]		
Relative	1.2 ± 0.3	1.2 ± 0.3			1.4 ± 0.4	1.4 ± 0.3			1.4 ± 0.3	1.5 ± 0.3			1.5 ± 0.3	1.5 ± 0.3		
	1.1–1.4	1.1–1.3	0.380	0.25	1.2–1.6	1.3–1.5	0.879	0.04	1.3–1.6	1.5–1.6	0.386	0.21	1.4–1.7	1.4–1.6	0.897	0.04
	[19]	[36]			[25]	[40]			[25]	[49]			[19]	[42]		
PROs, mean ± SD 95% CI [*n*]																
K‐SES_18_ present	4.3 ± 1.9	4.4 ± 1.9			6.0 ± 1.9	6.1 ± 1.7			8.4 ± 12.	8.0 ± 1.1			8.8 ± 1.4	8.4 ± 1.3		
	3.4–5.2	4.8–5.0	0.840	0.06	5.1–6.8	5.6–6.7	0.754	0.08	7.9–9.0	7.7–8.3	0.095	0.42	8.1–9.4	8.0–8.8	0.306	0.28
	[19]	[43]			[21]	[45]			[24]	[53]			[20]	[46]		
K‐SES_18_ future	7.9 ± 1.2	7.2 ± 1.7			7.8 ± 1.4	7.2 ± 1.6			8.1 ± 1.3	7.4 ± 1.5			8.1 ± 1.8	7.4 ± 1.7		
	7.3–8.5	6.6–7.7	0.099	0.46	7.1–8.4	6.7–7.7	0.166	0.37	7.6–8.7	7.0–7.8	0.054	0.43	7.2–8.9	6.9–7.9	0.171	0.37
	[19]	[43]			[21]	[45]			[24]	[53]			[20]	[46]		
									65.6 ± 17.9	53.5 ± 17.3			69.3 ± 19.7	59.8 ± 20.4		
ACL‐RSI	Not applicable				Not applicable				58.1–73.2	48.8–58.3	0.006*	0.69	59.1–77.5	53.7–65.9	0.123	0.42
									[24]	[53]			[20]	[45]		
PROs, Median (IQR) [*n*]																
Tegner, median (IQR)	2.0 (1.0)	2.0 (1.0)	0.809	‐	3.0 (2.0)	3.0 (1.0)	0.067	‐	5.0 (3.0)	4.0 (4.0)	0.046*	‐	7.5 (3.0)	7.0 (4.0)	0.295	‐
[*n*]	[19]	[43]			[21]	[45]			[25]	[53]			[20]	[46]		
RTS, *n* (%)	2 (11)	2 (5)	0.580	‐	1 (5)	7 (16)	0.419	‐	12 (48)	18 (34)	0.319	‐	15 (75)	32 (70)	0.772	‐
[*n*]	[19]	[43]			[21]	[45]			[25]	[53]			[20]	[46]		

Abbreviations: ACL, anterior cruciate ligament; ACL‐RSI, anterior cruciate ligament‐return to sport index; CI, confidence interval; GJH, generalised joint hypermobility; IQR, interquartile range; K‐SES_18_, 18 item version of knee self‐efficacy scale subscale; LSI, limb symmetry index; *n*, number of patients; PROs, RTS, return to sport; patient reported outcomes; SD, standard deviation; Tegner, Tegner activity scale.

* *p* < 0.05.

### Secondary outcomes

Table 2 presents the outcomes for psychological response and level of knee‐strenuous activity where patients in the second ACL group reported higher mean ACL‐RSI (65.6 ± 17.9 vs. 53.5 ± 17.3, *p* = 0.006, Cohen's *d* = 0.69), and a higher median present Tegner level (5.0, IQR: 3.0 vs. 4.0, IQR: 4.0, *p* = 0.046) at the 8‐month follow‐up compared to the control group. No other significant differences between groups were found at any other follow‐up.

### Sensitivity analysis

Files S1 and S2 present tables of results for the sensitivity analyses where no significant differences were found between patients who provided data for each specific outcome variable across all follow‐ups.

## DISCUSSION

### Main findings

The main findings of the present study were that patients with GJH who sustained a second ACL injury (1) showed no significant differences in terms of quadriceps and hamstring strength, (2) reported greater confidence, lower negative emotions, and lesser risk appraisal towards RTS at 8 months, (3) and reported greater knee‐strenuous activity at 8 months after primary ACL reconstruction compared to control group, that is, patients with GJH who did not sustain a second ACL injury. These findings partially confirmed our hypothesis, as patients in the second ACL injury group reported greater levels of knee‐strenuous activity, but showed similar muscle function symmetry compared to the control group. However, our hypothesis that the second ACL injury group would report similar psychological response compared to the control group was not confirmed.

### Muscle strength

There were no significant differences in muscle strength observed between the groups in the present study, irrespective of interpretation as strength in LSI or peak torque in relation to body weight. For the entire study population, patients achieved approximately 94% quadriceps LSI at 8 months and 98% quadriceps LSI at 12 months on average. To achieve symmetrical muscle strength, defined as LSI > 90% has previously been associated with reduced risk of a second ACL injury [15]. Conversely, achieving ≥90% quadriceps LSI 6 months after ACL reconstruction has been reported to increase odds of revision ACL reconstruction [11]. In a previous study from Project ACL [21], in patients with or without GJH who had RTS, there was no difference in knee muscle strength symmetry. In contrast, patients with GJH have been reported to exhibit lower muscle strength [19] and jumping capacity [31] compared to patients without GJH, although these studies did not analyse patients after ACL reconstruction. Recovery of muscle strength is an important factor of rehabilitation after ACL reconstruction to cope with the patient's desired knee‐strenuous activity, although this study does not imply that muscle strength is different in patients with GJH who sustain a second ACL injury compared with patients not sustaining a second ACL injury.

### PROs

Patients who sustained a second ACL injury scored higher on ACL‐RSI, exceeding the MIC threshold of 2.6 [32], 8 months after primary ACL reconstruction compared to patients with no second ACL injury. In addition, patients in the second ACL group reported a greater level of knee‐strenuous activity, although not a greater proportion of RTS, 8 months after ACL reconstruction, reaching the MDC threshold of 1 [9]. Previous studies have reported an association between greater scores on psychological PROs and the ability to RTS, that is, to participate in more knee‐strenuous activity [20, 30, 42]. Zarzycki et al. [43] also found that patients who sustained a second ACL injury scored greater on ACL‐RSI and passed a physical RTS test battery earlier compared to patients who did not sustain a second ACL injury. This may suggest that in patients who early on display high confidence, and low risk appraisal towards RTS, and reach rehabilitation milestones, a slower progression along the RTS continuum could be more important due to the probable elevated risk for a second ACL injury. For patients with GJH, this recommendation could be even more important as they are already predisposed to greater risk of a second ACL injury, although this needs to be confirmed with well‐designed research.

Previous literature has reported that RTS, that is, participating in knee‐strenuous activity, is associated with increased risk of a second ACL injury [14, 37], where patients who sustained a second ACL injury RTS 25 days earlier compared to patients who did not sustain a second ACL injury [27]. This study did not analyse the time to RTS as a factor for a second ACL injury, although the result found in our study indicates that patients who sustained a second ACL injury increased participation to a more knee‐strenuous level 8 months after primary ACL reconstruction compared to their counterparts who did not sustain a second ACL injury.

### Limitations

There are a few limitations to this study. First, although the number of comparisons performed in this study increases the risk of chance findings (type I error), no correction such as the Bonferroni correction was applied due to the possibility of type II errors. However, to strengthen the power of the results and to combat the risk chance findings, Cohen's *d* was added to the analysis. Second, the possible influence of associated injuries [44], or additional surgical procedures during ACL reconstruction [29], could not be analysed due to the lack of surgical data collected in Project ACL. Lastly, the level of knee‐strenuous activity was determined with Tegner which does not distinguish between level of competition, and exposure, which can be different between patients with or without a second ACL injury, affecting the risk of a second ACL injury. Taken together, the results of this study should be interpreted with caution.

## CONCLUSION

Patients with GJH who sustained a second ACL injury reported greater confidence, lower negative emotions, and lesser risk appraisal towards RTS, and reported greater levels of knee‐strenuous activity at 8 months after primary ACL reconstruction compared to matched patients who did not sustain a second ACL injury. These findings might suggest that patients who report greater psychological readiness to RTS, that is, greater confidence, lower negative emotions and lesser risk appraisal, at 8 months, need more cautious guidance when increasing knee‐strenuous activity.

## AUTHOR CONTRIBUTIONS

Jakob Lindskog drafted the initial version of the manuscript, performed analysis of data, has approved the final work for publication, and has agreed to be accountable for all aspects of the work. Axel Sundberg, Johan Högberg, Rebecca Hamrin Senorski and Ramana Piussi have contributed majorly during the analysis and interpretation of data, has made important contributions during the drafting of the work, has approved the final work, and has agreed to be accountable for all aspects of the work. Kristian Samuelsson and Roland Thomeé have contributed during the interpretation of data, made meaningful contributions during the final stages of manuscript drafting, has approved the final work, and has agreed to be accountable for all aspects of the work. Eric Hamrin Senorski has contributed majorly during the drafting of the manuscript, analysis and interpretation of data, is responsible for the design concept, has approved the final work, and has agreed to be accountable for all aspects of the work.

## CONFLICT OF INTEREST STATEMENT

Author Kristian Samuelsson reports a relationship with Getinge AB that includes: board membership. The remaining authors declare no conflicts of interest.

## ETHICS STATEMENT

The principles of the Helsinki declaration have been used as guidance in this study. Ethical approval has been obtained from the Swedish Ethical Review Authority (registration number: 2020‐02501), and the Regional Ethical Review Board in Gothenburg, Sweden (registration numbers: 265–13, T023–17). Data for this study are based on a rehabilitation registry project (Project ACL), where all patients have received written information and have given their informed consent in the research project.

## Supporting information

Supporting information.

Supporting information.

## Data Availability

The data that support the findings of this study are available on reasonable request from the corresponding author. The data are not publicly available due to privacy or ethical restrictions.
