# Supplementary material for: Greater psychological response and participation in knee‐strenuous activity 8 months after anterior cruciate ligament reconstruction in patients with generalised joint hypermobility who sustained a second anterior cruciate ligament injury: A cross‐sectional registry study
Source: J Exp Orthop. 2025 Jul 13;12(3):e70351. doi: 10.1002/jeo2.70351 (PMC12255941; doi:10.1002/jeo2.70351)
Supplement: Supplementary file 1 — Supporting information. [file JEO2-12-e70351-s002.docx]

**Online resource**

| Online resource 1 Sensitivity analysis of demographic data between patients who provide data for the outcomes at the 10-week, and 4-month follow-up versus patients who did not. | | | | | | | | | | | | | |  |
| --- | --- | --- | --- | --- | --- | --- | --- | --- | --- | --- | --- | --- | --- | --- |
| Group | 2^nd^ ACL  n=32 | | | Control  n=64 | | | 2^nd^ ACL  n=32 | | | Control  n=64 | | | |  |
| Follow-up | 10-week | | | | | | 4-month | | | | | | |  |
| Patient demographics | Dropout  n = 12 | No Dropout  n = 20 | p-value | Dropout  n = 20 | No Dropout  n = 44 | p-value | Dropout  n = 7 | No Dropout  n = 25 | p-value | Dropout  n = 16 | No Dropout  n = 48 | p-value |  |  |
| Patient sex, females, n (%) | 6 (50) | 12 (60) | 0.718 | 9 (45) | 31 (71) | 0.093 | 3 (43) | 15 (60) | 0.669 | 9 (56.3) | 31 (64.6) | 0.565 |  |  |
| Age, years mean ± SD | 19 ± 3 | 20 ± 3 | 0.838 | 19 ± 2 | 20 ± 3 | 0.105 | 20 ± 4 | 19 ± 3 | 0.827 | 19 ± 2 | 20 ± 3 | 0.581 |  |  |
| Height, cm mean ± SD | 176 ± 10 | 173 ± 7 | 0.247 | 174 ± 11 | 172 ± 8 | 0.421 | 175 ± 11 | 174 ± 8 | 0.663 | 174 ± 12 | 173 ± 8 | 0.690 |  |  |
| Weight, kg mean ± SD | 75 ± 11 | 70 ± 9 | 0.157 | 66 ± 11 | 70 ± 12 | 0.320 | 77 ± 11 | 70 ± 9 | 0.102 | 68 ± 13 | 69 ± 11 | 0.898 |  |  |
| Time from injury to ACL reconstruction, months median (IQR) | 4.8 (4.2) | 3.3 (3.4) | 0.213 | 3.8 (9.2) | 3.4 (3.4) | 0.061 | 5.4 (3.8) | 3.4 (3.3) | 0.194 | 3.5 (3.8) | 3.5 (3.5) | 0.710 |  |  |
| Knee HE, n (%) [n missing] | 10 (91) [1] | 13 (65) [0] | 0.203 | 13 (68) [1] | 34 (79) [1] | 0.521 | 6 (100) [1] | 17 (68) [0] | 0.298 | 12 (80) [1] | 35 (75) [1] | 1.000 |  |  |
| Pre-injury Tegner, median (IQR) | 8.5 (1.0) | 8.5 (2.0) | 0.441 | 9.0 (2.0) | 9.0 (1.0) | 0.781 | 8.0 (2.0) | 9.0 (2.0) | 0.257 | 9.0 (1.0) | 9.0 (1.0) | 0.805 |  |  |
| Graft choice, n (%)  HT  BPTB | 12 (100)  0 | 18 (90)  2 (10) | 0.516 | 20 (100)  0 | 40 (91)  4 (9) | 0.300 | 7 (100)  0 | 23 (92)  2 (8) | 1.000 | 16 (100)  0 | 44 (92)  4 (8) | 0.564 |  |  |
| BPTB = Bone-Patellar Tendon-Bone autograft, cm = centimeters, GJH = Generalized Joint Hypermobility, HE = Hyperextension, HT = Hamstrings Tendon autograft, IQR = Interquartile range, kg = kilogram, n = Number of patients, Tegner = Tegner Activity Scale, SD = Standard deviation.  Dropout was defined as a patient who did not provide any data for the follow-up. | | | | | | | | | | | | | |  |

| Online resource 2 Sensitivity analysis of demographic data between patients who provide data for the outcomes at the 8- and 12-month follow-up versus patients who did not. | | | | | | | | | | | | |  |
| --- | --- | --- | --- | --- | --- | --- | --- | --- | --- | --- | --- | --- | --- |
| Group | 2^nd^ ACL  n=32 | | | Control  n=64 | | | 2^nd^ ACL  n=32 | | | Control  n=64 | | |  |
| Follow-up | 8-month | | | | | | 12-month | | | | | |  |
| Patient demographics | Dropout  n = 6 | No Dropout  n = 26 | p-value | Dropout  n = 8 | No Dropout  n = 56 | p-value | Dropout  n = 12 | No Dropout  n = 20 | p-value | Dropout  n = 14 | No Dropout  n = 50 | p-value |  |
| Patient sex, females, n (%) | 3 (50) | 15 (58) | 1.000 | 4 (50) | 36 (64) | 0.460 | 7 (58) | 11 (55) | 1.000 | 8 (57) | 32 (64) | 0.757 |  |
| Age, years mean ± SD | 20 ± 4 | 19 ± 3 | 0.626 | 20 ± 3 | 19 ± 3 | 0.801 | 20 ± 3 | 19 ± 3 | 0.661 | 19 ± 2 | 20 ± 3 | 0.624 |  |
| Height, cm mean ± SD | 176 ± 6 | 174 ± 9 | 0.676 | 172 ± 8 | 173 ± 10 | 0.760 | 175 ± 7 | 174 ± 9 | 0.615 | 173 ± 8 | 173 ± 10 | 0.969 |  |
| Weight, kg mean ± SD | 72 ± 7 | 72 ± 10 | 0.962 | 67 ± 11 | 69 ± 12 | 0.605 | 72 ± 9 | 72 ± 10 | 0.945 | 68 ± 9 | 69 ± 12 | 0.873 |  |
| Time from injury to ACL reconstruction, months median (IQR) | 5.1 (27.9) | 3.4 (3.7) | 0.068 | 7.4 (9.0) | 3.4 (3.3) | 0.071 | 4.1 (7.7) | 4.0 (3.4) | 0.652 | 3.8 (5.0) | 3.4 (3.5) | 0.715 |  |
| Knee HE, n (%) [n missing] | 5 (83) [0] | 18 (72) [1] | 1.000 | 6 (75) [0] | 41 (76) [2] | 1.000 | 10 (91) [1] | 13 (65) [0] | 0.203 | 7 (54) [1] | 40 (82) [1] | 0.064 |  |
| Pre-injury Tegner, median (IQR) | 8.5 (2.0) | 9.0 (1.0) | 0.669 | 8.5 (1.0) | 9.0 (1.0) | 0.705 | 9.0 (2.0) | 8.5 (1.0) | 0.291 | 9.0 (2.0) | 9.0 (1.0) | 0.200 |  |
| Graft choice, n (%)  HT  BPTB | 6 (100)  0 | 24 (92)  2 (8) | 1.000 | 8 (100)  0 | 52 (93)  4 (7) | 1.000 | 12 (100)  0 | 18 (90)  2 (10) | 0.516 | 13 (93)  1 (7) | 47 (94)  3 (6) | 1.000 |  |
| BPTB = Bone-Patellar Tendon-Bone autograft, cm = centimeters, GJH = Generalized Joint Hypermobility, HE = Hyperextension, HT = Hamstrings Tendon autograft, IQR = Interquartile range, kg = kilogram, n = Number of patients, Tegner = Tegner Activity Scale, SD = Standard deviation.  Dropout was defined as a patient who did not provide any data for the follow-up. | | | | | | | | | | | | |  |
